# Effect of Algae and Plant Lectins on Planktonic Growth and Biofilm Formation in Clinically Relevant Bacteria and Yeasts

**DOI:** 10.1155/2014/365272

**Published:** 2014-05-28

**Authors:** Mayron Alves Vasconcelos, Francisco Vassiliepe Sousa Arruda, Victor Alves Carneiro, Helton Colares Silva, Kyria Santiago Nascimento, Alexandre Holanda Sampaio, Benildo Cavada, Edson Holanda Teixeira, Mariana Henriques, Maria Olivia Pereira

**Affiliations:** ^1^Biologically Active Molecules Laboratory (Biomol-Lab), Department of Biochemistry and Molecular Biology, Federal University of Ceará, Fortaleza 60440-970, CE, Brazil; ^2^Institute for Biotechnology and Bioengineering (IBB), Center of Biological Engineering, University of Minho, 4710-057 Braga, Portugal; ^3^Integrated Laboratory of Biomolecules (LIBS), Department of Pathology and Legal Medicine, Faculty of Medicine, Federal University of Ceará, Fortaleza 60430-160, CE, Brazil

## Abstract

This study aimed to evaluate the abilities of plant and algae lectins to inhibit planktonic growth and biofilm formation in bacteria and yeasts. Initially, ten lectins were tested on *Staphylococcus epidermidis, Staphylococcus aureus, Klebsiella oxytoca, Pseudomonas aeruginosa, Candida albicans*, and *C. tropicalis* at concentrations of 31.25 to 250 **μ**g/mL. The lectins from *Cratylia floribunda* (CFL), *Vatairea macrocarpa* (VML), *Bauhinia bauhinioides* (BBL), *Bryothamnion seaforthii* (BSL), and *Hypnea musciformis* (HML) showed activities against at least one microorganism. Biofilm formation in the presence of the lectins was also evaluated; after 24 h of incubation with the lectins, the biofilms were analyzed by quantifying the biomass (by crystal violet staining) and by enumerating the viable cells (colony-forming units). The lectins reduced the biofilm biomass and/or the number of viable cells to differing degrees depending on the microorganism tested, demonstrating the different characteristics of the lectins. These findings indicate that the lectins tested in this study may be natural alternative antimicrobial agents; however, further studies are required to better elucidate the functional use of these proteins.

## 1. Introduction 

Microorganisms are able to grow and adhere to many surfaces, forming complex communities known as biofilms. In biofilms, cells grow in multicellular aggregates that are encased in a self-produced extracellular matrix [1]. Moreover, it is known that these microcommunities exhibit distinct phenotypes with respect to gene transcription, growth rate, and enhanced resistance to antimicrobial agents [2–4].

Biofilms have been found to be involved in many chronic diseases, such as chronic otitis, tonsillitis, cystic fibrosis, periodontal diseases, and urinary tract infections [5–7]. Furthermore, biofilms can form on medical implants such as catheters, artificial hips, and contact lenses, and, owing to their increased resistance to antimicrobial agents, these infections can often only be treated by removing the device [8, 9]. Compared to their planktonic counterparts, the microorganisms in biofilms are less sensitive to biocides and antibiotics, complicating the management of device-associated infections and limiting therapeutic options [10, 11]. Thus, the discovery of new compounds that are able to eradicate such biofilms is critical. In fact, in recent years, lectins have been demonstrated to be active compounds against bacteria and fungi in both planktonic cells and biofilms [12–14].

Lectins are a class of carbohydrate-binding proteins of nonimmune origin which are organized into closely structurally related families [15]. Although lectins were first found in plants, they occur in all classes and families of organisms, from bacteria and viruses to mammals [16–18]. They are responsible for deciphering sugar codes through complex surface interactions and play a central role in a number of biological processes, such as infections, cell communication, and cell growth [19, 20]. Based on their capacity to bind and recognize specific carbohydrates, lectins are involved in several biological activities [21–25]. Although lectins have sequence homology and structural similarities, they differ in their various biological properties [26]. Thus, it is important to identify new lectins with biotechnological potential as antimicrobial and antibiofilm agents.

Lectins can recognize and reversibly bind to carbohydrates on cell surfaces and interact with cell wall polysaccharides and/or glycoconjugates in the cell membrane [27, 28]. Moreover, lectins can interact with bacterial lipopolysaccharides (LPS) or with the extracellular matrix of microorganisms [29–31]. Some lectins have also shown antimicrobial activity and are able to interfere with the formation of biofilms [14, 32–36].

Thus, this work aimed to evaluate the* in vitro* antimicrobial activity of a set of plant and red algae lectins towards clinically relevant microorganisms, including bacteria and yeast.

## 2. Materials and Methods

### 2.1. Microorganisms

The microorganisms used in this study included gram-positive bacteria:* Staphylococcus epidermidis* CECT231 and* Staphylococcus aureus* JKD 6008, gram-negative bacteria:* Klebsiella oxytoca* ATCC13182 and* Pseudomonas aeruginosa* ATCC 10145, and yeasts:* Candida albicans* ATCC90028 and* Candida tropicalis* ATCC750, which were obtained from the Colección Española de Cultivos Tipo (CECT) and the American Type Culture Collection (ATCC). The* S. aureus *strain JKD6008 is a clinical isolate that has developed resistance to vancomycin (vancomycin-intermediate* S. aureus* (VISA)) [37].

### 2.2. Lectins Purification

The plant and algae lectins were isolated by different chromatographic techniques including affinity and ion exchange chromatographies. The references for purification are described as follows:* Dioclea megacarpa* lectin (DML) [38],* Dioclea rostrata* lectin (DRL) [39],* Dioclea guianensis* lectin (DGL) [40],* Dioclea violacea* lectin (DVL) [41],* Cratylia floribunda* lectin (CFL) [42],* Vatairea macrocarpa* lectin (VML) [43],* Bauhinia bauhinioides *lectin (BBL) [44],* Luetzelburgia auriculata* agglutinin (LAA) [45],* Bryothamnion seaforthii* lectin (BSL) [46], and* Hypnea musciformis* lectin (HML) [47].

### 2.3. Culture Conditions

The bacteria were grown in Trypticase Soy Agar medium (TSA, Liofilchem, Italy) and incubated at 37°C for 24 h. After growth on solid medium, an isolated colony was removed and inoculated into 10 mL of Trypticase Soy Broth (TSB, Liofilchem, Italy) and incubated for 18 h at 37°C under constant agitation at 120 rpm. Prior to use, the cell concentration of each inoculum was adjusted to 2 × 10^6^ cells/mL using a spectrophotometer (620 nm) and the calibration curves previously determined for each bacterium. The yeast culture conditions were the same as those previously mentioned; however, the media included Sabouraud Dextrose Agar and Broth (SDA and SDB, respectively; Liofilchem, Italy), and the concentration of each yeast inoculum was adjusted to 2 × 10^6^ cells/mL using a Neubauer chamber.

### 2.4. Planktonic Growth Assays

The effects of lectins on planktonic growth were determined by the broth microdilution method in 96-well polystyrene plates. Briefly, lectins were diluted in culture medium (SDB for yeasts and TSB for bacteria) to concentrations of 31.25 to 250 *μ*g/mL, and 100 *μ*L of each lectin was incubated with 100 *μ*L of each microorganism (previously adjusted to 2 × 10^6^ cells/mL). In order to provide a negative control, similar assays were performed without lectins. All plates were incubated at 37°C for 24 h at 120 rpm, and optical density of each well was recorded at 640 nm (OD_640_) using an automated microplate reader (Synergy TM HT Multidetection Microtiter Reader). The inhibition of planktonic growth by lectins was classified as weak growth inhibition (10–40% of inhibition), medium growth inhibition (40–80% of inhibition), or strong growth inhibition (80–100% of inhibition).

### 2.5. Biofilm Assays

Bacterial and yeast biofilms were grown in microtiter plates as previously described by Stepanovic et al. [48], with some modifications. Briefly, lectins at concentrations ranging from 31.25 to 250 *μ*g/mL were added to the plates containing microorganisms at 2 × 10^6^ cells/mL. The plates were then incubated on a horizontal shaker (120 rpm) at 37°C for 24 h for biofilm development. Afterwards, the content of each well was removed, and biofilms were washed twice with 200 *μ*L of well sterilized water to remove weakly adherent cells. In order to remove biofilm-entrapped bacterial cells, 200 *μ*L of sterile water was added to each well and plates were placed in an ultrasonic bath operating at 50 kHz for 6 min (Sonicor Instruments, Copiague, NY, USA). For the yeast biofilms, each well was scraped vigorously with a pipette tip. Both procedures were previously well established in the laboratory. Serial decimal dilutions from the obtained suspensions were plated on TSA or SDA (depending on the microorganism) to verify the number of viable cells in the biofilms. The agar plates were then incubated for 24 h at 37°C, and the total number of colony-forming units (CFU) per unit area (log CFU/cm^2^) of the well was counted.

The biofilm biomass was quantified by crystal violet staining method. For the fixation of biofilms, 200 *μ*L of 99% methanol (Romil, UK) was added to each well, and after 15 min the methanol was removed and the plates were allowed to dry at 25°C. Then, 200 *μ*L of crystal violet stain (Merck, Germany) was added to each well. After 5 min, the unbound dye was removed and wells washed twice with water. Finally, 200 *μ*L of acetic acid (33%, v/v) (Pronalab, Portugal) was added to all wells to dissolve the crystal violet stain, and the optical density was measured at 570 nm (OD_570_).

### 2.6. Statistical Analysis

Statistical analyses were performed by GraphPad Prism version 5.0 software from Microsoft Windows. The data from all the assays were compared using one-way analysis of variance (ANOVA) with the Bonferroni post hoc test. *P* < 0.01 was considered to be statistically significant.

## 3. Results 

### 3.1. Lectin Purification

**Figure 1:**
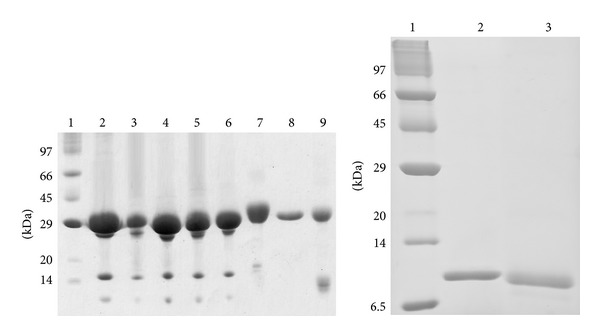
SDS-polyacrylamide gel electrophoresis (15%). (a) Plant lectins. Line 1: Molecularmass markers; line 2: DML; line 3: DRL; line 4: DGL; line 5: DVL; line 6: CFL; line 7: VML; line 8: BBL and line 9: LAA. (b) Algae lectins. Line 1: Molecular mass markers; line 2: BSL and line 3: HML.

The purities of the lectins were determined by SDS-PAGE, as shown in Figure 1. The Diocleinae lectins displayed a pattern of subunits characteristic of the lectins from that subtribe. The proteins migrated as three bands, consisting of the full-length intact polypeptide chain (*α*-chain) and two fragments, *β* and *γ*. VML migrated as four bands, two major bands (double bands) of approximately 34 and 32 kDa (*α*-chains) and two minor bands of 22 and 13 kDa (the *β*- and *γ*-chains, resp.). LAA migrated as two major bands of 29 kDa (double bands) and two minor bands at approximately 15 kDa. A different pattern was observed for BBL, which exhibited only one band of approximately 32 kDa. Regarding the algae lectins, BSL and HML exhibited only one band of approximately 10 kDa. Furthermore, all lectins showed hemagglutinating activity against rabbit erythrocytes and were fully inhibited by 100 mM D-glucose (Diocleinae lectins), 100 mM D-galactose (VML, LAA, and BBL), and the glycoprotein porcine stomach mucin (algae lectins).

### 3.2. Effect of Lectins on Planktonic Growth

From all the lectins tested, only 5 (CFL, VML, BBL, HML, and BSL) presented any effect against one or more of the microorganisms. Although these 5 plant and algae lectins were not effective against all bacteria and yeasts, they were all selected for the biofilm assays (Table 1).

CFL at 250 *μ*g/mL moderately inhibited the planktonic growth of* S. aureus*; however, the lowest concentrations of this protein showed weak inhibition. CFL also weakly reduced the growth of* S. epidermidis* at 250 and 125 *μ*g/mL. CFL was not able to reduce the growth of the gram-negative bacteria. For the yeasts, CFL moderately inhibited* C. albicans* at all concentrations, but no effect on* C. tropicalis* was observed.

Both of the D-galactose-specific lectins (VML and BBL) caused some reduction in the planktonic growth of the microorganisms. VML at 250 *μ*g/mL completely inhibited the planktonic growth of* S. aureus* and* S. epidermidis*. VML was also able to inhibit, albeit weakly, the growth of* P. aeruginosa* and* C. albicans*. No effect of BBL was observed against* S. epidermidis*, and only weak inhibition of the other microorganisms was observed.

The algae lectins HML and BSL caused weak growth reductions in* S. aureus*,* S. epidermidis,* and* P. aeruginosa*. Only HML reduced the growth of* K. oxytoca*. Neither of the algae lectins reduced the yeast growth.

### 3.3. Effect of Lectins on Biofilm Mass and the Number of CFUs

**Figure 2:**
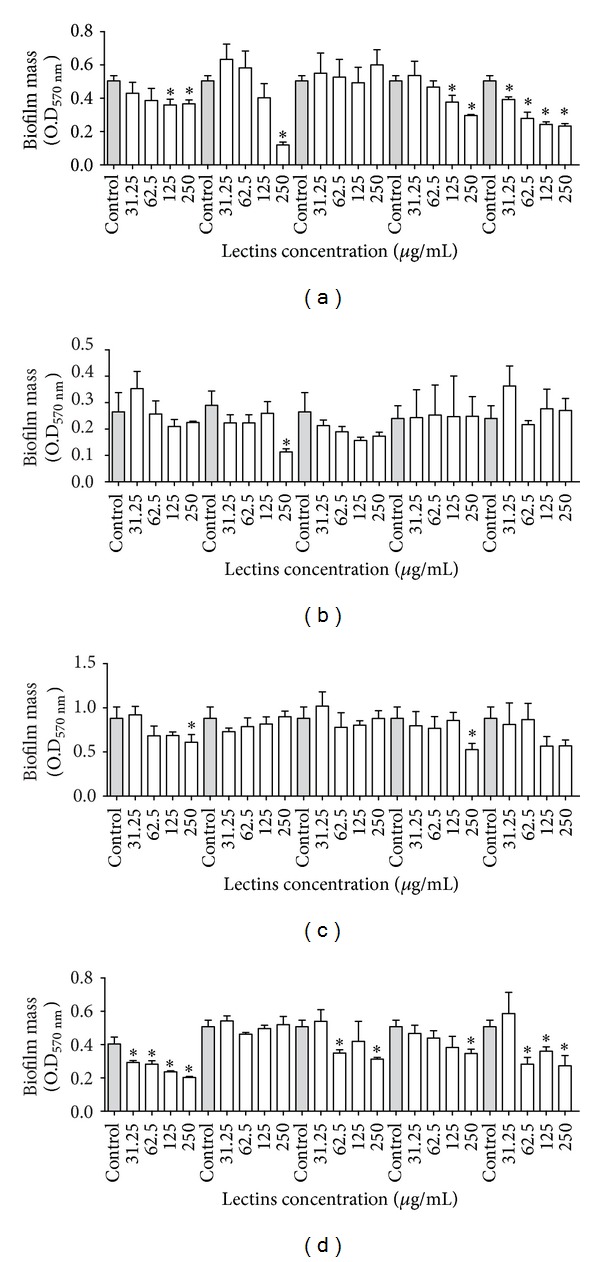
Effects of lectins on the biofilm biomasses of* Staphylococcus aureus* JKD6008 (a);* Staphylococcus epidermidis* CECT231 (b);* Pseudomonas aeruginosa* ATCC10145 (c) and* Klebsiella oxytoca* ATCC13182 (d). *P < 0.01 relative to control.

**Figure 3:**
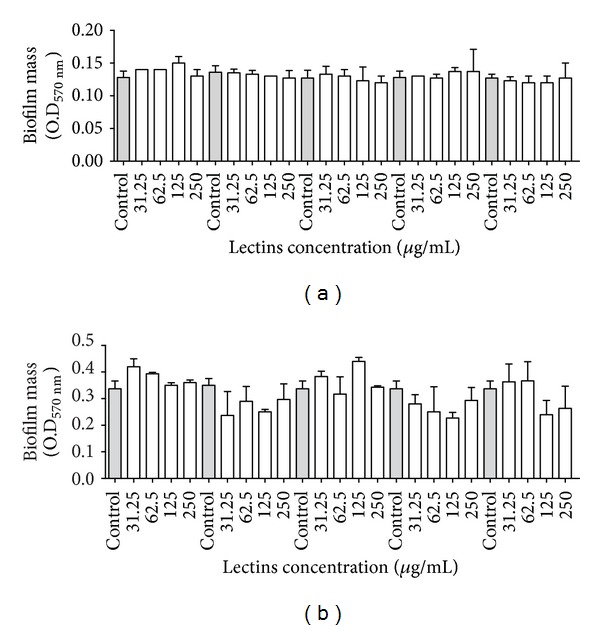
Effects of lectins on the biofilm biomasses of* Candida albicans* ATCC90028 (a) and* Candida tropicalis* ATCC750 (b). *P < 0.01 relative to control.

**Figure 4:**
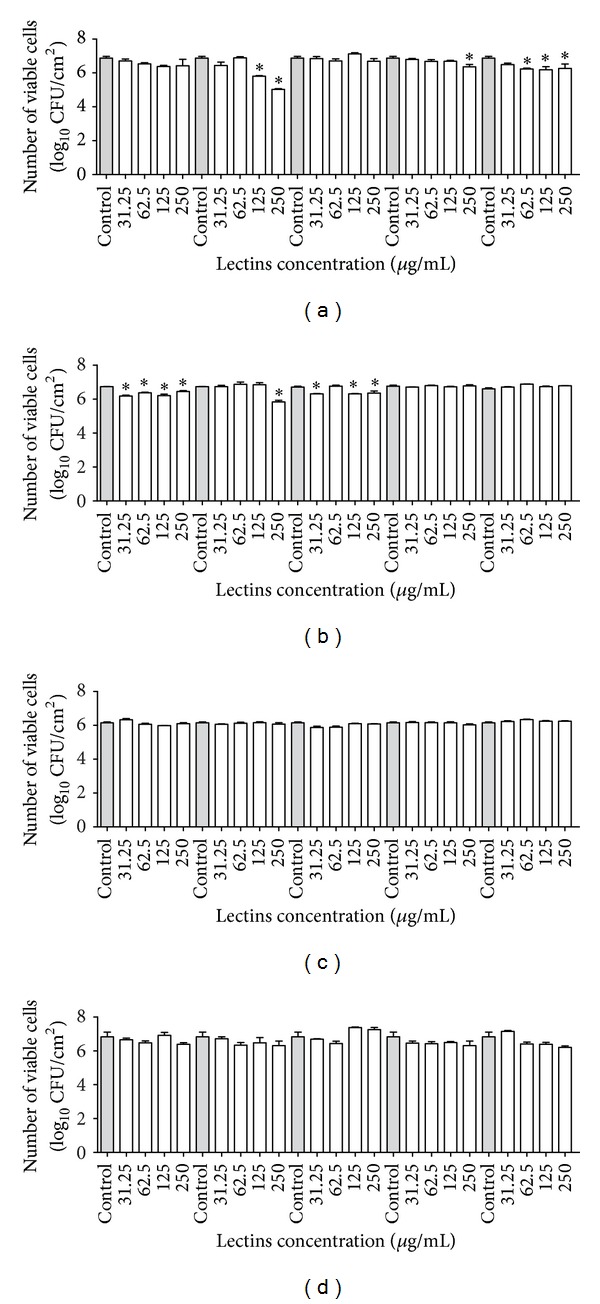
Effects of lectins on the number of viable cells in the biofilms of* Staphylococcus aureus* JKD6008 (a);* Staphylococcus epidermidis* CECT231 (b);* Pseudomonas aeruginosa* ATCC10145 (c) and* Klebsiella oxytoca* ATCC13182 (d). *P < 0.01 relative to control.

**Figure 5:**
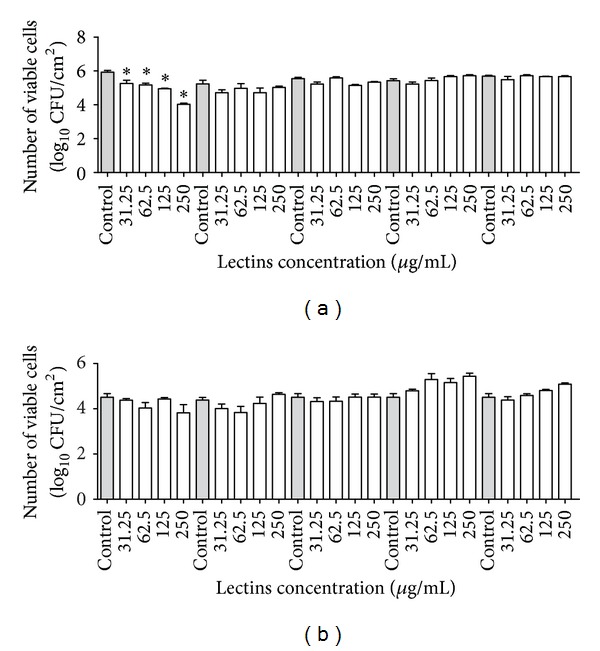
Effects of lectins on the number of viable cells in the biofilms of* Candida albicans* ATCC90028 (a) and* Candida tropicalis* ATCC750 (b). *P < 0.01 relative to control.

The effects of the lectins on the biomass and the number of biofilm viable cells are shown in Figures 2, 3, 4, and 5. Interestingly, the lectins were able to prevent the establishment of biomass in the biofilms of the bacteria whose planktonic growth was also sensitive to the lectins. The plant lectins CFL and VML were able to inhibit the biomass formation of* S. aureus* in biofilms (Figure 2(a)), but only VML was able to influence biofilm formation in* S. epidermidis* at the concentration of 250 *μ*g/mL (Figure 2(b)). BSL could decrease the biofilm mass of* S. aureus* at all concentrations (Figure 2(a)); however, HML only caused a small decrease at the highest concentrations (Figure 2(a)). Regarding the gram-negative bacteria, CFL, BBL, and the algae lectins were able to reduce the biomass of* K. oxytoca* to different extents (Figure 2(d)). At 250 *μ*g/mL, CFL and HML reduced the biomass of the* P. aeruginosa* biofilm (Figure 2(c)). Although some lectins inhibited the planktonic growth of the yeasts, no lectin was able to reduce the yeast biofilm mass (Figure 3).

After incubating the microorganisms with the lectins for 24 hours, the number of cells was evaluated and expressed as log CFU/cm^2^ (Figures 4 and 5). Similar to the reduction of biomass observed for* S. aureus* and* S. epidermidis*, a decrease in the number of viable cells in the biofilm was observed in the presence of the highest concentration of VML (Figure 4(a)). Moreover, HML and BSL caused a small decrease in the number of viable cells of* S. aureus* (Figure 4(a)), as did CFL and BBL for* S. epidermidis* (Figure 4(b)). Interestingly, the lectins were unable to decrease the number of viable cells of gram-negative bacteria (Figures 4(c) and 4(d)). Regarding the yeasts, only CFL was able to decrease the number of viable cells in the* C. albicans* biofilms (Figure 5(a)).

## 4. Discussion 

This work investigated the effect of plant and algae lectins on planktonic growth and biofilm formation in both bacteria and yeast. The observed effects of VML on the planktonic growth of* S. aureus*,* S. epidermidis*, and the gram-positive bacteria agreed with the results of other studies [13, 49, 50], which have reported that the lectins isolated from the seeds of* Eugenia uniflora* (EuniSL) and from the heartwood of* Myracrodruon urundeuva* display activity against* S. aureus* at low concentrations of 1.5 and 0.58 *μ*g/mL, respectively. On the other hand, Costa et al. [51] demonstrated that the* Phthirusa pyrifolia* leaf lectin (PpyLL) has an MIC at 250 *μ*g/mL to* S. epidermidis*, while it does not inhibit the growth of* S. aureus*. Regarding the gram-negative bacteria, the lectins tested in this study had weak or no effects on planktonic growth (Table 1). The above-referenced EuniSL and the lectin from* M. urundeuva* exhibited MICs of 5 and 4.68 *μ*g/mL to* P. aeruginosa*, respectively, while PpyLL did not have any activity against this bacterium [49–51]. Moreover, the lectin isolated from the seaweed* Solieria filiformis* [52] reduced the planktonic growth of* P. aeruginosa*, as well as that of other species of gram-negative bacteria, by approximately 25%, but only at concentrations of 1 mg/mL. These differences in the concentrations of the lectins on the reduction of bacterial growth most likely resulted from differences in the strains tested or in the sugar specificity of the different lectins.

The mechanisms by which lectins exert their activity are not well described, but it is believed that their antibacterial activity against gram-positive and gram-negative bacteria occurs through interactions of the lectins with components of the bacterial cell wall, including teichoic and teichuronic acids, peptidoglycans, and lipopolysaccharides [12, 53].

Interestingly, the lectins of the genus* Dioclea* and CFL are homologous, but only CFL reduced the planktonic growth of the microorganisms. These lectins belong to the subtribe Diocleinae and have a high degree of structural similarity and the same specificity [29]. Despite their high similarity, these lectins induce different responses in biological assays [54, 55]. According to Cavada et al. [26], the differences in biological activity among Diocleinae lectins may result from small changes in the relative orientations of their carbohydrate-binding sites, their binding specificity for complex carbohydrates, or their pH-dependent oligomerization state. These factors may explain the observation that only CFL reduced the growth of the microorganisms, while the homologous lectins exhibited no such activity (Table 1).

Despite displaying identical sugar specificity (D-galactose), VML and BBL also showed different activities against the growth of the microorganisms (Table 1), and their different characteristics may account for their different activities, such as fine variations in their sugar specificities. Furthermore, VML was presented as an N-glycoprotein [56]; however, Silva et al. [44] showed that BBL is not a glycoprotein using sugar analysis.

In this study, the D-galactose-specific lectins VML and BBL were able to weakly reduce the growth of* C. albicans* (Table 1). According to Santana et al. [57], the lectin isolated from* Opuntia ficus* cladodes is able to affect the growth of* C. albicans*, reducing fungal growth by approximately 59%. According to Wong et al. [36], lectins do not directly inhibit fungal growth by changing the structure and/or permeability of the fungal membrane but rather by indirect effects produced by the binding of the lectins to carbohydrates on the surface of the fungal cell wall. On the other hand, Melo et al. [58] showed that the lectin isolated from* Luetzelburgia auriculata* (LAA) inhibits the fungal growth of* Saccharomyces cerevisiae*. Using transmission electronic microscopy, the authors observed the presence of lectins in the outer portion of the cell wall and in the inner surface of the yeast cell wall bordering the cell membrane. Moreover, their results suggested that the lectin can interfere with the intracellular transport of protons to the external environment [58].

In general, the different lectins were able to reduce the biomass of some bacteria, often in a concentration-dependent manner (Figure 2). For planktonic growth, VML at 250 *μ*g/mL was able to reduce the biomass of* S. aureus* and* S. epidermidis*. CFL also showed a reduction in the biomass of* K. oxytoca* and* P. aeruginosa*, but BBL only showed a reduction in* K. oxytoca*. VML was more effective at 250 *μ*g/mL (Figure 2). Cavalcante et al. [55] also showed that the lectin isolated from the seeds of* Canavalia maritima* (ConM) was able to reduce the biomass of* Streptococcus mutans* biofilms when applied at a concentration of 200 *μ*g/mL, which is a concentration similar to that used in this work.

Some lectins isolated from algae have also shown activity against bacterial biofilm formation. For example, lectins from the red algae* Bryothamnion triquetrum* and* Bryothamnion seaforthii* were able to attach to the acquired pellicle and reduce the adherence of streptococci strains at 100 *μ*g/mL [33].

Lectins can cause bacterial aggregation [51, 59–61]. Liljemark et al. [62] suggested that the formation of large aggregates causes a decrease in the number of adherent bacteria, which may explain why some of the lectins tested in this study inhibited biofilm formation instead of reducing bacterial growth. Moreover, some studies have indicated that biofilm formation is directly related to the type of LPS produced by the bacteria [63]. Because lectins are able to interact with bacterial LPS [30, 31], the abilities of some lectins to inhibit biofilm formation in gram-negative bacteria may result from interactions between the lectin and LPS, affecting the adherence of these bacteria and thus influencing biofilm formation.

Interestingly, the lectins used in this study reduced the number of viable cells in only the gram-positive bacteria, and only CFL was able to decrease the number of viable cells of* C. albicans* (Figure 2). Most likely, the reduced number of viable cells in the biofilms of* S. aureus* and* S. epidermidis* by VML and in those of* C. albicans* by CFL was caused by the inhibition of the planktonic growth of the microorganisms. Lakhtin et al. [35] showed that lectins of bifidobacterial species and lactobacillus displayed destructive properties towards* C. albicans* and* S. aureus* biofilms. According to that author, in the presence of the lectins,* C. albicans* and* S. aureus* biofilm degradation occurs in sequential steps, including biofilm refinement, the appearance of edge cavities, segmentation, the detachment of fragments, and, finally, lysis [35].

In summary, some of the lectins tested here were able to inhibit planktonic growth and/or biofilm formation in the microorganisms. Moreover, the most promising lectins showed differences in activity, which can be explained by the different characteristics of these proteins. On the basis of these findings, further studies will be required to investigate the probable mechanisms of action of these lectins against microorganisms as well as search for new antimicrobial lectins.

## Figures and Tables

**Table 1:** Inhibition of planktonic growth by lectins from *Cratylia  floribunda* (CFL), *Vatairea macrocarpa* (VML), *Bauhinia bauhinioides* (BBL), *Bryothamnion  seaforthii* (BSL), and *Hypnea  musciformis* (HML).

	CFL				VML				BBL				HML				BSL			
Concentration (*μ*g/mL)	250	125	62.5	31.25	250	125	62.5	31.25	250	125	62.5	31.25	250	125	62.5	31.25	250	125	62.5	31.25
*Staphylococcus aureus *	++	+	+	+	+++	+	+	+	+	+	ND	ND	+	+	+	ND	+	ND	ND	ND
*Staphylococcus epidermidis *	+	+	ND	ND	+++	ND	ND	ND	ND	ND	ND	ND	+	ND	ND	ND	+	+	ND	ND
*Pseudomonas aeruginosa *	ND	ND	ND	ND	+	+	+	+	+	+	+	+	+	+	+	+	+	+	+	+
*Klebsiella oxytoca *	ND	ND	ND	ND	ND	ND	ND	ND	+	+	+	ND	+	+	+	ND	ND	ND	ND	ND
*Candida albicans *	++	++	++	++	+	+	+	+	+	+	+	ND	ND	ND	ND	ND	ND	ND	ND	ND
*Candida tropicalis *	ND	ND	ND	ND	+	ND	ND	ND	+	ND	ND	ND	ND	ND	ND	ND	ND	ND	ND	ND

ND: not detected.

+: weak growth inhibition.

++: medium growth inhibition.

+++: strong growth inhibition.
